# Performance of the Abbott RealTime MTB and RIF/INH resistance assays for the detection of *Mycobacterium Tuberculosis* and resistance markers in sputum specimens

**DOI:** 10.1371/journal.pone.0251602

**Published:** 2021-05-12

**Authors:** Bezalem Tesfaye Araya, Kirubel Eshetu Ali, Dereje Assefa Geleta, Saba Gebremichael Tekele, Kassu Desta Tulu

**Affiliations:** 1 Department of Immunology, Armaur Hanson Research Institute, Addis Ababa, Ethiopia; 2 Ethiopian Public Health Institute, Addis Ababa, Ethiopia; 3 International Organization for Migration, Addis Ababa, Ethiopia; 4 Department of Medical Laboratory Sciences, College of Health Sciences, Wollo University, Dessie, Ethiopia; 5 Department of Medical Laboratory Sciences, College of Health Sciences, Addis Ababa University, Addis Ababa, Ethiopia; The University of Georgia, UNITED STATES

## Abstract

**Background:**

The Abbott RealTime MTB is an assay for the detection of *Mycobacterium tuberculosis (*MTB) complex DNA from respiratory specimens in combination with the Abbott RealTime RIF/INH assay for the detection of genetic resistance markers for isoniazid (INH) and rifampicin (RIF) from MTB positive isolates. Hence, this study aimed to evaluate the performance of the Abbott RealTime MTB and RIF/INH assays.

**Methods:**

A cross-sectional study was conducted on 289 study subjects presumptive to have pulmonary tuberculosis at Nigist Eleni Mohammed Memorial Hospital, South Ethiopia from April 2017 to June 2018. Two morning expectorated sputum specimens were collected from each study participant. One sample was tested directly by Xpert MTB/RIF assay at Nigist Eleni Mohammed Memorial Hospital and the other sample was used for smear microscopy, TB culture, Abbott RealTime MTB, and Abbott RealTime INH/RIF assays at International Clinical Laboratories, Addis Ababa, Ethiopia. The diagnostic performance of the Abbott RealTime MTB and INH/RIF assays were calculated against MGIT liquid culture and phenotypic drug susceptibility testing (DST) as the gold standard.

**Results:**

For the detection of MTB the Abbott RealTime MTB assay exhibited sensitivity 92.4% (95% CI 83.6–96.9), specificity 95.4% (95% CI 91.1–97.7), PPV 89.0% (95% CI 79.7–94.5) and NPV 96.9% (95% CI 93.0–98.7). For the detection of RIF resistance MTB, Abbott RealTime MTB RIF/INH concurred with phenotypic DST and Xpert MTB/RIF, while for the detection of INH resistance MTB, the sensitivity, specificity, PPV and NPV of the Abbott MTB RIF/INH assay was 84.2% (95% CI 60.4–96.6), 100% (95% CI 89.7–100), 100% and 91.9% (95% CI 80.0–96.9), respectively.

**Conclusions:**

The Abbott RTMTB and RIF/INH assays revealed high sensitivity and specificity in MTB diagnosis and provided reliable INH and RIF resistance profiles. This assay has a similar diagnostic performance to the Xpert MTB/RIF assay with the advantages of high-throughput.

## Background

Tuberculosis (TB) is the ninth leading cause of death worldwide and the leading cause from a single infectious agent. In 2018, an estimated 10.0 million incident cases and about half a million rifampicin-resistant TB (RR-TB) cases were reported worldwide. Ethiopia is one of the 30 high TB burden countries with an estimated 165,000 new cases of TB and 710 incidences of MDR (multidrug resistance)/RR-TB [1]. To meet the END TB strategy [2], early diagnosis and successful treatment of people with TB are crucial; however, there are still large and persistent gaps in case finding and treatment [3].

Molecular tests such as NAATs (Nucleic acid amplification tests) are the most promising tools to close this diagnostic gap since it provides faster results compared to culture and higher sensitivity compared to smear microscopy [4]. Because of the increasing use of NAATs and the potential impact on patient care and public health, the Center for Disease Control and Prevention (CDC) endorsed these tests to be performed on at least one respiratory specimen from each suspected TB cases and for whom the test result would change case management or TB control activities [5].

The Abbott m2000^™^ RT is a fully automated polymerase chain reaction (PCR) system with precise and proven performance for the diagnosis of infectious diseases such as HIV/ AIDS. Recently this platform integrates an assay for the qualitative detection of *Mycobacterium tuberculosis* (MTB) targeting *IS6110* and PAB genes from sputum, bronchoalveolar lavage (BAL), or N-acetyl-L-Cysteine (NALC)-treated sediments of sputum or BAL samples [6].

The Abbott Realtime MTB RIF/INH assay is a companion assay to the Abbott Realtime MTB; for the detection of RIF and/or INH resistance MTB targeting *rpoB*, and *inhA*, *katG* genes from MTB positive samples [7].

In this study, we evaluated the diagnostic performance of the Abbott RealTime MTB and Abbott RealTime MTB RIF/INH assays from sputum specimens using MGIT liquid culture (BACTEC MGIT 960; Becton Dickinson & Co., Franklin Lakes, NJ, USA), and first-line MGIT DST (Bactec MGIT 960 SIRE kit; Becton Dickinson & Co., NJ, USA) as a reference.

## Materials and methods

A cross-sectional study was conducted at Nigist Eleni Mohammed Memorial Hospital, South Ethiopia, from April 2017 to June 2018. Adults (≥18 years) with respiratory symptoms suggestive of MTB who can give sputum specimens were informed about the study. Using consecutive sampling technique a total of 289 participants were enrolled in this study. Persons on anti-TB treatment for more than two weeks were excluded from the study. Two morning expectorated sputum specimens with a minimum volume of 2-3ml were collected on two consecutive days from each study participant. The first collected fresh sputum specimen was tested by the Xpert MTB/RIF assay at Nigist Eleni Mohammed Memorial Hospital. The other specimen collected on the study participant’s second visit was transported to International Clinical laboratories; a laboratory accredited by Joint Commission International (JCI) on the same day of collection and processed the next day.

### Testing

#### Sample decontamination

N-acetyl-L-Cysteine (NALC)-NaOH solution in a volume equal to the specimen was added which allows its digestion and decontamination. This mixture was incubated for 15–20 minutes with vortexing for 15–20 seconds. Phosphate buffer (pH 6.8) was added up to the top ring on the centrifuge tube and then centrifuged at a speed of 3000 g for 15–20 minutes in a refrigerated centrifuge. The supernatant was decanted into a suitable container and the sediment was resuspended using 2 ml PBS.

#### AFB smears

The resuspended sediment after specimen decontamination was used to make a smear on a glass slide. The slides were stained using Ziehl-Neelsen (ZN) staining then observed under a light microscope.

#### MTB culture

For liquid culture 0.8 ml of MGIT growth supplement/PANTA and 0.5 ml of a well-mixed processed/concentrated specimen were added to MGIT tube and loaded to Bactec MGIT 960 mycobacterial detection system. The instrument reports a tube negative if it remains negative for 42 days [8]. MGIT positive tubes were visually inspected for contamination (turbidity), tubes that appeared turbid with the presence of non-acid fast bacteria on ZN staining were reported as “tube contaminated”. For AFB positive tubes identification was performed using an Immunochromatographic test kit (SD MPT64TB Ag). The leftover processed sediment of sputum after TB culture and AFB smear was stored at -20°C for later analysis using the Abbott RealTime MTB assay.

#### First-line MGIT DST

Susceptibility testing with the MGIT system was performed with MGIT cultures that tested positive at least 3 days but no more than 5 days after 1:5 dilution following the manufacturer instructions. For each appropriately labeled MGIT tube 800 μl SIRE Supplement, 100 μl of drug concentrations (0.1μg/ml critical concentration for INH, 1.0 μg/ml critical concentration for RIF) and 500 μl processed specimen was added. All tubes were mixed gently and loaded into MGIT 960 instrument using the MGIT carrier. The instrument continuously monitors the tubes for 4 to 21 days. Results were interpreted automatically and reported as susceptible (S) or resistant (R) [9].

#### Xpert MTB/RIF assay

The sample reagent (2ml) supplied with the test was added in a 2:1 ratio to the sputum (1ml). The mixture was mixed vigorously and incubated at room temperature for 15 minutes. Two ml of the reagent sample mix was then transferred to an Xpert MTB/RIF cartridge using a pasteur pipette and the cartridge was loaded onto Xpert MTB/RIF instrument after appropriate patient data is filled into the software [10].

#### Abbott RealTime MTB assay

Inactivation reagent (IR), which inactivates the MTB viability, was added at a ratio of 3:1 to NALC-treated sediments of sputum and incubated for 1–24 hours under class II biological safety cabinet (BSC). The Abbott m2000sp instrument was used for the extraction of DNA followed by the sample preparation step. At the end of sample preparation, the amplification master mix was loaded onto an m2000sp which dispenses 25μl aliquots of the master mix followed by 25μl aliquots of the extracted eluates to a 96-well optical reaction plate. The plate was sealed manually and transferred to the m2000rt for amplification and detection. Results were reported as “MTB not detected” or “MTB detected” [6].

#### Abbott RealTime MTB RIF/INH assay

The RT MTB RIF/INH assay was performed as a standalone test; IR treated specimens (22 MTB positive specimens), assay controls (Abbott RealTime MTB RIF/INH positive and negative controls), and sample preparation reagents were loaded on an m2000sp. After the sample preparation steps were completed, m2000sp dispense 25μl aliquots of master mixes and 25μl of extracted sample eluates to each master mix in a 96 well optical reaction plate. The plate is manually sealed and transferred to an Abbott m2000rt for amplification and detection. The result was interpreted according to the manufacturer’s instruction [7].

### Data quality assurance

To generate quality and reliable data, all quality control checks were done before, during, and after data collection. Moreover, all laboratory assays were done by maintaining quality control procedures following Standard Operating Procedures (SOPs). The Abbott RealTime MTB RIF/INH positive and negative controls were used to verify the test result. To avoid biased interpretation of test results the personnel performing the sputum specimen for TB culture, Abbott RealTime MTB, Xpert MTB/RIF were blinded to the results of the other tests.

### Statistical analysis

The data were imported and analyzed by STATA statistical software version 12.1 (StataCorp LLC, College Station, TX, USA). Sensitivity, specificity, positive, and negative predictive values with 95% CI were calculated using liquid culture and phenotypic DST as a reference. *P-*values less than 0.05 were considered significant.

### Ethical considerations

The study was conducted after obtaining ethical clearance from the research and ethical review committee of the Department of Medical Laboratory Sciences, School of Allied Health Sciences, College of Health Sciences; Addis Ababa University. An official permission letter was obtained from the study site. Moreover, written informed consent was obtained from each study participant before data collection.

## Results

A total of 289 paired sputum specimen was collected from each study participants, of which 10 contaminated culture result, 3 Xpert MTB/RIF invalid result and 2 invalid Abbott MTB assay result was excluded from the analysis. Statistical analysis was performed on 274 valid results, 19.6% (54/274) were smear and culture positive (SPCP), 9.1% (25/274) were smear-negative culture-positive (SNCP), 70.3% (194/274) were smear and culture-negative (SNCN) while 0.4% (1/274) was smear-positive culture-negative (SPCN).

### Performance of Abbott RealTime MTB assay for detection of MTB

Table 1 shows the performance of the Abbott RealTime MTB and Xpert MTB/RIF assays. The Abbott RealTime MTB assay identified MTB correctly in 73 (92.4%) out of 79 bacteriological confirmed TB cases. Overall using MGIT liquid culture media as a reference the sensitivity of this assay was 96.3% (95% CI, 91.2–100%) from smear-positive and 84% (95% CI: 63.1–94.7%) from smear-negative specimens. MTB was not detected in 186 out of 195 (95.4%) MTB negative specimens, while 9 (4.6%) positives were reported from culture-negative cases resulting in a specificity of 95.4 (95% CI: 91.1–97.7). The other pair of 274 specimens was tested by Xpert MTB/RIF assay. The diagnostic sensitivities of this test were 94.4% (95% CI: 87.5–100%) from smear-positive and 56% (95% CI 30.0–82.0) from smear-negative specimens, while the specificity was 96.9 (95% CI: 93.1–98.7) as compared to liquid culture.

**Table 1:** Performance of Abbott RealTime MTB and Xpert MTB/RIF assays for MTB detection using MGIT liquid culture as a reference (n = 274).

		Sensitivity (95% CI)	Specificity (95% CI)	PPV (95% CI)	NPV (95% CI)
Abbott RealTime MTB	Culture positive (n = 79)	92.4 (83.6–96.9)	N/A	N/A	N/A
	Smear + (n = 54)	96.3 (86.2–99.4)	N/A	N/A	N/A
	Smear—(n = 25)	84 (63.1–94.7)	N/A	N/A	N/A
	Culture negative (n = 195)	N/A	95.4 (91.1–97.7)	89.0 (79.7–94.5)	96.9 (93.0–98.7)
Xpert MTB/RIF	Culture positive (n = 79)	82.3 (71.7–89.6)	N/A	N/A	N/A
	Smear + (n = 54)	94.4 (83.6–98.5)	N/A	N/A	N/A
	Smear—(n = 25)	56.0 (35.3–74.9)	N/A	N/A	N/A
	Culture negative (n = 195)	N/A	96.9 (93.1–98.7)	91.5 (81.9–96.5)	93.1 (88.5–96.0)

CI-Confidence Interval, PPV-Positive Predictive Value, NPV-Negative Predictive Value, N/A-Not applicable

### Performance of Abbott MTB RIF/INH assay

A total of 66 MTB positive NALC/NaOH pellets of sputum were processed by Abbott RIF/INH assay for the qualitative detection of RIF and INH resistance MTB. Incomplete or missing resistance profiles were observed in 13 (19.7%) isolates due to signals for one or more targets probe(s) were below the limit of detection (LOD) and only 53 (80.3%) interpretable results were compared with indirect DST. These comprise 10 MDR, 9 INH mono-resistance, 2 RIF mono-resistance, and 32 pan-susceptible TB specimens.

Out of 19 (35.8%) INH resistance specimens, the Abbott RealTime MTB RIF/INH assay detected 16 (84.2%) of them while discordance was reported with 3 (15.8%) samples, which tested susceptible with the Abbott RealTime MTB RIF/INH assay but INH resistant by phenotypic DST. In all 34 (64.1%) INH susceptible samples, no mutations were detected in INH resistance determining regions as shown in Table 2. For INH resistance mutation detection the sensitivity for the detection of INH TB resistance was 84.2% (95% CI: 59.5–95.8), specificity was 100% (95% CI: 87.4–100).

**Table 2:** Performance of Abbott RealTime MTB INH/RIF assay for the detection of MDR-TB using phenotypic DST as a reference (n = 53).

Indirect DST						
	Isoniazid			Rifampicin		
	Resistant		Susceptible	Resistant	Susceptible	
Abbott RealTime RIF/INH	Resistant	16	0	12	0	
	Susceptible	3	34	0	41	
Sensitivity = 84.2% (95%CI 59.5–95.8)				Sensitivity: 100% (95%CI 69.9–100)		
Specificity = 100% (95%CI 87.4–100)				Specificity = 100% (95%CI 89.3–100)		
PPV = 100% (95%CI 75.9–100)				PPV = 100% (95%CI 69.9–100)		
NPV = 91.9% (95%CI 76.9–97.9)				NPV = 100% (95%CI 89.3–100)		
Xpert				12		0
MTB/RIF				0		41
				Sensitivity: 100% (95%CI 69.9–100)		
				Specificity: 100% (95%CI 69.9–100)		
				PPV: 100% (95%CI 69.9–100)		
				NPV: 100% (95%CI 69.9–100)		

PPV-Positive Predictive Value, NPV-Negative Predictive Value.

In the case of RIF resistance, no discrepant result was observed for both Abbott RealTime RIF/INH and Xpert MTB/RIF assays as compared with phenotypic DST.

## Discussion

In this study, we evaluated the performance of the Abbott RealTime MTB and Abbott RealTime RIF/INH assay in a resource-limited setting with a high burden of TB for the first time. In this study, the assay performed well against Xpert MTB/RIF and MGIT liquid culture for the detection of MTB from smear-negative and smear-positive samples. The results of our study are comparable to a meta-analysis which includes 11 studies that reported the pooled sensitivity of 96% (95% CI 88–99) and a pooled specificity of 97% (95% CI 96.0–99.0%) for the Abbott Realtime MTB assay [11].

The sensitivity of the Abbott RealTime MTB assay for the detection of MTB among smear-negative specimens was higher (84%) than the Xpert/RIF assay (56%), in general, the Abbott RealTime MTB assay detected 28% (7/25) more SNCP cases than the Xpert MTB/RIF assay. This finding is in line with the study by Scott L et al conducted in high TB and HIV co-infected settings [12]. This could be due to the Abbott RealTime MTB assay uses two target genes for the detection of MTB: multi-copy insertion sequence (IS6110) and the single-copy protein antigen b (PAB), while the Xpert MTB/RIF assay amplifies a portion of the *rpoB* gene containing the 81 base pair "core" region. Studies have shown that the diagnostic performance of PCR for TB can be improved with the use of more than one target gene [13–15], this is because there are strains of MTB lacking IS6110 or PAB [16–18]. The other possible cause for the higher sensitivity of Abbott RT assay could be the difference in samples, concentrated sputum samples after TB culture was used for Abbott RealTime assay while raw sputum specimens were used for the Xpert MTB/RIF assay.

The results obtained from the Abbott RealTime MTB RIF/INH assay showed no discrepant results for RIF resistance detection as compared to the indirect DST and the Xpert MTB/RIF assay, this finding is in line with a study conducted by Hoffman-Thiel et al. [19]. But in 3/19 INH resistance specimens, mutation(s) was not detected with the Abbott RealTime MTB RIF/INH assay resulting with 84.2% sensitivity. This proportion is relatively similar to the study conducted by Ruiz P *et al* (78.8%) [20] and Tam KK *et al* (84%) [21]. Discordant INH resistance pattern between DST and this assay could be as a result of INH resistance is linked with multiple genes and appears more complex [22]. Mutations in the *katG* and the *inhA* genes, which are target genes for Abbott INH/RIF assay, are associated with only approximately about 80% of INH-resistant MTB isolates [23], other genes such as *ahpC*, *kasA*, *oxyR*-*ahpC*, and *furA*-*katG* which are not detected with this test are also related with INH resistance [22].

Detecting both RIF and INH resistance at the same time, the Abbott RealTime MTB RIF/INH assay has an advantage over Xpert MTB/RIF assay to detect INH mono-resistance MTB. In this study three (6.8%) INH mono-resistance MTB was detected which were disregarded by Xpert MTB/RIF assay. It has been reported that INH mono-resistance TB has been associated with treatment failure with a standard 6-month first-line regimen [24, 25], so early identification of these cases may help to reduce poor treatment outcomes.

One of the limitations of the Abbott RealTime MTB RIF/INH assay found in this study was higher (19.7%) invalid results reported, as the target probe signals could not be detected from INH and RIF resistance determining probes. Higher results were reported in a study conducted by Scott L et al (33%) [12] and in China by Tam KK et al (30.9%) [21]. In this study 76.9% (10/13) of indeterminate results were from smear-negative samples (*p*<0.05), suggesting the “Below LOD” report may be due to the paucibacillary nature of samples.

The Abbott m2000 automated platform (Abbott, Des Plains, Illinois, USA) is well established for HIV viral load testing in many settings, including Ethiopia, with trained staff and quality procedures already in place. The Abbott RealTime MTB assay was easy to perform since the process is automated with very little hands-on time required after specimen preparation. In our study, including specimen inactivation, a full RealTime MTB detection run (93 specimens and 3 controls) can be completed in about 9.5 hours, which may help for the increased need to rapidly test higher numbers of specimens.

There are some limitations to our study: i) we have done a single liquid culture on the specimen that is collected on the participant’s second visit, the type of sample was not random. ii) We were unable to use the same specimen for all tests; the Abbott RealTime MTB assay was done on decontaminated and frozen specimens, while the Xpert MTB/RIF assay was done on raw and fresh specimens collected on the participant’s first visit. iii) it was beyond the scope of our study to include more MTB isolates with relevant mutations and to resolve discrepant results for isoniazid resistance MTB isolates using sequencing. However, this should be considered in future researches.

## Conclusions

The Abbott RealTime MTB assay has high sensitivity and specificity in detecting MTB from smear-positive and smear-negative respiratory specimens, besides the Abbott RealTime MTB RIF/INH assay provides a reliable drug resistance profile directly from sputum specimens. Interestingly this test has a similar diagnostic performance to Xpert MTB/RIF assay with the advantages of high-throughput and simultaneously diagnosis of INH and RIF resistance MTB.

## Supporting information

S1 Data Raw data of the study. (PDF)

## Abbreviations

INH: isoniazid
NAATs: Nucleic acid amplification tests
NPV: Negative predictive value
PAB: protein antigen b
PCR: Polymerase Chain Reaction
PPV: positive predictive value
RIF: rifampicin
TB: Tuberculosis

## References

[1] World Health Organization. Global tuberculosis report, 2019. Geneva, Switzerland: WHO; 2019. https://www.who.int/tb/publications/global_report/en/. Accessed July 12, 2020.

[2] World Health Organization. 2015. The end TB strategy: global strategy and targets for tuberculosis prevention, care and control after 2015. World Health Organization, Geneva, Switzerland. http://www.who.int/tb/strategy/End_TB_Strategy.pdf. Accessed 20 December 2016.

[3] World Health Organization. Global tuberculosis report, 2018. Geneva, Switzerland: WHO; 2018. https://apps.who.int/iris/handle/10665/274453

[4] Caulfield AJ, Wengenack NL. Diagnosis of active tuberculosis disease: From microscopy to molecular techniques. *J ClinTuberc Other Mycobact Dis*. 2016;4:33–43. 10.1016/j.jctube.2016.05.005 31723686 PMC6850262

[5] Centers for Disease Control and Prevention. Updated guidelines for the use of nucleic acid amplification tests in the diagnosis of tuberculosis. Morbidity and mortality weekly report. 2009.16;58(1):7–10. Available from: https://www.cdc.gov/mmwr/preview/mmwrhtml/mm5801a3.htm. Accessed June 30, 2020. 19145221

[6] Tang N, Frank A, Pahalawatta V, et al. Analytical and clinical performance of Abbott RT MTB, an assay for detection of *Mycobacterium tuberculosis* in pulmonary specimens. *Tuberculosis*. 2015;95(5):613–619. 10.1016/j.tube.2015.05.010 26119174

[7] Kostera J, Leckie G, Tang N, et al. Analytical and clinical performance characteristics of the Abbott RealTime MTB RIF/INH Resistance, an assay for the detection of rifampicin and isoniazid-resistant *Mycobacterium tuberculosis* in pulmonary specimens. *Tuberculosis*. 2016;101:137–143. 10.1016/j.tube.2016.09.006 27865383

[8] Siddiqi S. H., Ruesch-Gerdes S. MGIT procedure manual for BACTEC MGIT 960 TB system. Becton Dickinson, Franklin Lakes, NJ. 2006

[9] Becton, Dickinson and Company. BACTEC MGIT-960 SIRE kit for the antimycobacterial susceptibility testing of *Mycobacterium tuberculosis*. Becton, Dickinson & Company, Sparks, MD, USA.

[10] World Health Organization. Xpert MTB/RIF: WHO policy update and Implementation manual. http://www.who.int/tb/laboratory/xpert_launchupdate/en/.Accessed 16 May 2018.

[11] Wang M., Xue M., Wu S., Zhang M., Wang Y., Liu Q., et al. Abbott RealTime MTB and MTB RIF/INH assays for the diagnosis of tuberculosis and rifampicin/isoniazid resistance. *Infection*, *Genetics and Evolution*, 2019;71:54–59. 10.1016/j.meegid.2019.03.012 30902741

[12] Scott L, David A, Noble L, Nduna et al. Performance of the Abbott RealTime MTB and MTB RIF/INH assays in a setting of High Tuberculosis and HIV coinfection in South Africa. *J ClinMicrobiol*. 2017;55(8):2491–2501. 10.1128/JCM.00289-17 PMC5527428 28592547

[13] Sarokhalil DD, Fooladi A, Bameri Z, Nasiri M, Feizabadi M. Cytochrome CYP141: a new target for direct detection of *Mycobacterium tuberculosis* from clinical specimens. *ActamicrobiologicaetimmunologicaHungarica*. 2011;58(3):211–217. 10.1556/AMicr.58.2011.3.4 21983322

[14] Narayanan S, Parandaman V, Narayanan P, et al. Evaluation of PCR using TRC4 and IS6110 primers in detection of tuberculous meningitis. *J ClinMicrobiol*. 2001;39(5):2006–2008. 10.1128/JCM.39.5.2006-2008.2001 PMC88071 11326036

[15] Palanisamy G, Aggarwal S, Sharma K, Saibaba B, Dhillon MS. Improved diagnostic potential of polymerase chain reaction by amplification of multiple gene targets in osteoarticular tuberculosis. *J Med Sci*. 2017;37(4):163.

[16] Huyen MN, Tiemersma EW, Kremer K, et al. Characterisation of *Mycobacterium tuberculosis* isolates lacking IS6110 in Viet Nam. *Int J Tuberc Lung Dis*. 2013;17(11):1479–1485. 10.5588/ijtld.13.0149 24125454

[17] Howard ST, Oughton MT, Haddad A, Johnson WM. Absence of the genetic marker IS6110 from a strain of *Mycobacterium tuberculosis* isolated in Ontario. *Can J Infect Dis Med Microbiol*.1998;9(1):48–53. 10.1155/1998/292491 22346535 PMC3250867

[18] Chauhan DS, Sharma VD, Parashar D, Chauhan A. Molecular typing of *Mycobacterium tuberculosis* isolates from different parts of India based on IS6110 element polymorphism using RFLP analysis. *Indian J Med Res*. 2007;125(4):577. 17598945

[19] Hofmann-Thiel S, Molodtsov N, Antonenka U, Hoffmann H. Evaluation of the Abbott RealTime MTB and RT MTB INH/RIF Assays for direct detection of *Mycobacterium tuberculosis* complex and resistance markers in respiratory and extrapulmonary specimens. *J ClinMicrobiol*. 2016;54(12):3022–3027. 10.1128/JCM.01144-16 27733630 PMC5121395

[20] Ruiz P CM, Vaquero M, Gutierrez JB, Casal M. Evaluation of a new automated Abbott RealTime MTB RIF/INH assay for qualitative detection of rifampicin/isoniazid resistance in pulmonary and extra-pulmonary clinical samples of *Mycobacterium tuberculosis*. *Infect Drug Resist*. 2017;10:463–467. 10.2147/IDR.S147272 29263682 PMC5724413

[21] Tam KK-G, Leung KS-S, To SW-C, et al. Direct detection of *Mycobacterium tuberculosis* and drug resistance in respiratory specimen using Abbott RealTime MTB detection and RIF/INH resistance assay. *DiagnMicrobiol Infect Dis*. 2017;89(2):118–124. 10.1016/j.diagmicrobio.2017.06.018 28780247

[22] Palomino JC, Martin A. Drug resistance mechanisms in *Mycobacterium tuberculosis*. *Antibiotics*. 2014;3(3):317–340. 10.3390/antibiotics3030317 27025748 PMC4790366

[23] Seifert M, Catanzaro D, Catanzaro A, Rodwell TC. Genetic mutations associated with isoniazid resistance in *Mycobacterium tuberculosis*: a systematic review. *PloS One*. 2015;10(3):e0119628. 10.1371/journal.pone.0119628 25799046 PMC4370653

[24] Villegas L, Otero L, Sterling TR, et al. Prevalence, risk factors, and treatment outcomes of isoniazid-and rifampicin-mono-Resistant Pulmonary Tuberculosis in Lima, Peru. *PloS One*. 2016;11(4):e0152933. 10.1371/journal.pone.0152933 27045684 PMC4821555

[25] Jacobson KR, Theron D, Victor TC, Streicher EM, Warren RM, Murray MB. Treatment outcomes of isoniazid-resistant tuberculosis patients, Western Cape Province, South Africa. *Clin Infect Dis*. 2011;53(4):369–372. 10.1093/cid/cir406 21810750 PMC3202325

