# Supplementary material for: Performance of the Abbott RealTime MTB and RIF/INH resistance assays for the detection of Mycobacterium Tuberculosis and resistance markers in sputum specimens
Source: PLoS One. 2021 May 12;16(5):e0251602. doi: 10.1371/journal.pone.0251602 (PMC8115802; doi:10.1371/journal.pone.0251602)
Supplement: S1 Data — (PDF) [file pone.0251602.s001.pdf]

Laboratory results excluding invalid results and contaminated culture results				
**Sample ID**	**MTB culture result**	**Smear result**	**Abbott RealTime MTB**	**Xpert MTB/RIF**
BT 001	Positive	Negative	Positive	Positive
BT 002	Positive	Positive	Positive	Positive
BT 003	Positive	Negative	Positive	Positive
BT 004	Positive	Positive	Positive	Positive
BT 005	Negative	Negative	Negative	Negative
BT 006	Negative	Negative	Negative	Negative
BT 007	Positive	Positive	Positive	Positive
BT 008	Positive	Negative	Positive	Positive
BT 009	Positive	Negative	Positive	Negative
BT 010	Positive	Positive	Positive	Positive
BT 012	Negative	Negative	Negative	Negative
BT 013	Negative	Negative	Negative	Negative
BT 014	Negative	Negative	Negative	Negative
BT 015	Negative	Negative	Negative	Negative
BT 016	Negative	Negative	Negative	Negative
BT 017	Positive	Negative	Positive	Positive
BT 018	Positive	Positive	Positive	Positive
BT 020	Positive	Positive	Positive	Positive
BT 021	Negative	Negative	Negative	Negative
BT 022	Negative	Negative	Negative	Negative
BT 023	Positive	Positive	Positive	Positive
BT 024	Negative	Negative	Negative	Negative
BT 025	Negative	Negative	Negative	Negative
BT 026	Negative	Negative	Negative	Negative
BT 027	Negative	Negative	Negative	Negative
BT 028	Positive	Negative	Positive	Negative
BT 029	Positive	Negative	Negative	Negative
BT 030	Positive	Positive	Positive	Positive
BT 031	Negative	Negative	Negative	Negative
BT 032	Negative	Negative	Negative	Negative
BT 033	Positive	Positive	Negative	Negative
BT 034	Positive	Positive	Negative	Negative

BT 035	Negative	Negative	Negative	Negative
BT 036	Negative	Negative	Negative	Negative
BT 037	Positive	Positive	Positive	Positive
BT 038	Positive	Positive	Positive	Positive
BT 039	Negative	Negative	Negative	Negative
BT 040	Negative	Negative	Negative	Negative
BT 041	Positive	Positive	Positive	Positive
BT 042	Positive	Positive	Positive	Positive
BT 043	Positive	Negative	Positive	Negative
BT 044	Positive	Negative	Positive	Negative
BT 045	Negative	Negative	Negative	Negative
BT 046	Negative	Negative	Negative	Negative
BT 047	Positive	Positive	Positive	Positive
BT 048	Positive	Positive	Positive	Positive
BT 049	Negative	Negative	Negative	Negative
BT 050	Negative	Negative	Negative	Negative
BT 051	Positive	Positive	Positive	Positive
BT 052	Negative	Negative	Negative	Negative
BT 053	Negative	Negative	Negative	Negative
BT 054	Positive	Positive	Positive	Positive
BT 055	Negative	Negative	Negative	Negative
BT 056	Negative	Negative	Negative	Negative
BT 057	Negative	Negative	Negative	Negative
BT 058	Positive	Negative	Positive	Positive
BT 059	Negative	Negative	Negative	Negative
BT 060	Positive	Positive	Positive	Positive
BT 061	Positive	Positive	Positive	Positive
BT 062	Positive	Positive	Positive	Positive
BT 063	Negative	Negative	Negative	Negative
BT 064	Negative	Negative	Negative	Negative
BT 065	Negative	Negative	Negative	Negative
BT 066	Positive	Positive	Positive	Positive
BT 067	Positive	Positive	Positive	Positive
BT 068	Negative	Negative	Negative	Negative

BT 069	Negative	Negative	Negative	Negative
BT 070	Positive	Negative	Positive	Positive
BT 071	Positive	Positive	Positive	Positive
BT 072	Negative	Negative	Negative	Negative
BT 073	Negative	Negative	Negative	Negative
BT 074	Negative	Negative	Negative	Negative
BT 075	Positive	Positive	Positive	Positive
BT 076	Positive	Positive	Positive	Positive
BT 077	Negative	Negative	Negative	Negative
BT 078	Negative	Negative	Negative	Negative
BT 079	Positive	Positive	Positive	Positive
BT 080	Negative	Negative	Negative	Negative
BT 081	Negative	Negative	Negative	Negative
BT 082	Negative	Negative	Negative	Negative
BT 083	Negative	Negative	Negative	Negative
BT 084	Positive	Positive	Positive	Positive
BT 085	Positive	Negative	Positive	Positive
BT 086	Positive	Positive	Positive	Positive
BT 087	Negative	Negative	Negative	Negative
BT 088	Positive	Positive	Positive	Positive
BT 089	Positive	Positive	Positive	Positive
BT 090	Negative	Negative	Negative	Negative
BT 091	Negative	Negative	Negative	Negative
BT 092	Negative	Negative	Negative	Negative
BT 093	Negative	Negative	Negative	Negative
BT 094	Positive	Positive	Positive	Positive
BT 095	Negative	Negative	Negative	Negative
BT 096	Negative	Negative	Negative	Negative
BT 097	Negative	Negative	Negative	Negative
BT 098	Positive	Positive	Positive	Positive
BT 099	Negative	Negative	Negative	Negative
BT 100	Negative	Negative	Negative	Negative
BT 101	Positive	Positive	Positive	Positive
BT 102	Negative	Negative	Negative	Negative

BT 103	Negative	Negative	Negative	Negative
BT 104	Negative	Negative	Negative	Negative
BT 105	Positive	Positive	Positive	Positive
BT 106	Negative	Negative	Negative	Negative
BT 107	Negative	Negative	Negative	Negative
BT 108	Negative	Negative	Negative	Negative
BT 109	Negative	Negative	Negative	Negative
BT 110	Negative	Negative	Negative	Negative
BT 111	Positive	Negative	Negative	Negative
BT 112	Negative	Negative	Negative	Negative
BT 113	Negative	Negative	Negative	Negative
BT 114	Positive	Positive	Positive	Positive
BT 115	Positive	Positive	Positive	Negative
BT 116	Negative	Negative	Negative	Negative
BT 117	Negative	Negative	Negative	Negative
BT 118	Negative	Negative	Negative	Negative
BT 119	Negative	Negative	Negative	Negative
BT 120	Negative	Negative	Negative	Negative
BT 121	Positive	Positive	Positive	Positive
BT 122	Positive	Positive	Positive	Positive
BT 123	Negative	Negative	Negative	Negative
BT 124	Negative	Negative	Negative	Negative
BT 125	Negative	Negative	Negative	Negative
BT 126	Negative	Negative	Negative	Negative
BT 127	Positive	Positive	Positive	Positive
BT 128	Negative	Negative	Negative	Negative
BT 130	Negative	Negative	Negative	Negative
BT 131	Negative	Negative	Negative	Negative
BT 132	Negative	Negative	Negative	Negative
BT 133	Negative	Negative	Negative	Negative
BT 134	Negative	Negative	Negative	Negative
BT 136	Positive	Positive	Positive	Positive
BT 137	Negative	Negative	Negative	Negative
BT 138	Negative	Negative	Negative	Negative

BT 139	Negative	Negative	Negative	Negative
BT 140	Negative	Negative	Negative	Negative
BT 141	Negative	Negative	Negative	Negative
BT 142	Negative	Negative	Negative	Negative
BT 143	Negative	Negative	Negative	Negative
BT 144	Negative	Negative	Negative	Negative
BT 145	Negative	Negative	Negative	Negative
BT 147	Negative	Negative	Negative	Negative
BT 148	Negative	Negative	Negative	Negative
BT 149	Negative	Negative	Negative	Negative
BT 150	Negative	Negative	Negative	Negative
BT 151	Negative	Negative	Negative	Negative
BT 152	Positive	Positive	Positive	Positive
BT 155	Positive	Positive	Positive	Positive
BT 156	Negative	Negative	Negative	Negative
BT 157	Negative	Negative	Negative	Negative
BT 158	Positive	Positive	Positive	Positive
BT 159	Positive	Positive	Positive	Positive
BT 160	Negative	Negative	Negative	Negative
BT 161	Negative	Negative	Negative	Negative
BT 162	Negative	Negative	Negative	Negative
BT 164	Negative	Negative	Negative	Negative
BT 165	Negative	Negative	Negative	Negative
BT 166	Negative	Negative	Negative	Negative
BT 169	Negative	Negative	Negative	Negative
BT 170	Positive	Positive	Positive	Positive
BT 171	Negative	Negative	Negative	Negative
BT 172	Negative	Negative	Negative	Negative
BT 173	Negative	Negative	Negative	Negative
BT 174	Negative	Negative	Negative	Negative
BT 177	Negative	Negative	Negative	Negative
BT 178	Negative	Negative	Negative	Negative
BT 179	Positive	Positive	Positive	Positive
BT 180	Negative	Negative	Negative	Negative

BT 181	Negative	Negative	Negative	Negative
BT 183	Negative	Negative	Negative	Negative
BT 184	Negative	Negative	Negative	Negative
BT 185	Positive	Positive	Positive	Positive
BT 186	Positive	Positive	Positive	Positive
BT 187	Negative	Negative	Negative	Negative
BT 188	Negative	Negative	Negative	Negative
BT 189	Negative	Negative	Negative	Negative
BT 190	Negative	Negative	Negative	Negative
BT 191	Negative	Negative	Negative	Negative
BT 193	Negative	Negative	Negative	Negative
BT 194	Negative	Negative	Negative	Negative
BT 195	Negative	Negative	Negative	Negative
BT 196	Negative	Negative	Negative	Negative
BT 197	Positive	Positive	Positive	Positive
BT 198	Negative	Negative	Negative	Negative
BT 199	Negative	Negative	Negative	Negative
BT 200	Negative	Negative	Negative	Negative
BT 201	Negative	Negative	Negative	Negative
BT 202	Negative	Negative	Negative	Negative
BT 203	Positive	Negative	Positive	Positive
BT 204	Positive	Positive	Positive	Positive
BT 205	Positive	Positive	Positive	Positive
BT 206	Negative	Negative	Negative	Negative
BT 207	Negative	Negative	Negative	Negative
BT 208	Negative	Negative	Negative	Negative
BT 209	Positive	Negative	Positive	Positive
BT 210	Negative	Negative	Negative	Negative
BT 211	Negative	Negative	Negative	Negative
BT 212	Negative	Negative	Negative	Negative
BT 213	Negative	Negative	Negative	Negative
BT 214	Negative	Negative	Negative	Negative
BT 215	Positive	Negative	Positive	Positive
BT 216	Negative	Negative	Negative	Negative

BT 217	Negative	Negative	Negative	Negative
BT 218	Negative	Negative	Negative	Negative
BT 219	Negative	Negative	Negative	Negative
BT 220	Negative	Negative	Negative	Negative
BT 221	Negative	Negative	Negative	Negative
BT 222	Positive	Negative	Negative	Negative
BT 223	Negative	Negative	Negative	Negative
BT 224	Negative	Negative	Negative	Negative
BT 225	Negative	Negative	Negative	Negative
BT 226	Negative	Negative	Negative	Negative
BT 227	Negative	Negative	Negative	Negative
BT 228	Negative	Negative	Negative	Negative
BT 229	Negative	Negative	Negative	Negative
BT 230	Negative	Negative	Negative	Negative
BT 231	Negative	Negative	Negative	Negative
BT 232	Negative	Negative	Negative	Negative
BT 233	Negative	Negative	Negative	Negative
BT 234	Positive	Negative	Positive	Negative
BT 235	Negative	Negative	Negative	Negative
BT 236	Negative	Negative	Negative	Negative
BT 237	Negative	Negative	Negative	Negative
BT 238	Negative	Negative	Negative	Negative
BT 239	Negative	Negative	Negative	Negative
BT 240	Negative	Negative	Negative	Negative
BT 241	Negative	Negative	Negative	Negative
BT 242	Negative	Negative	Negative	Negative
BT 243	Negative	Negative	Negative	Negative
BT 244	Negative	Negative	Negative	Negative
BT 245	Negative	Negative	Negative	Negative
BT 246	Negative	Negative	Negative	Negative
BT 247	Negative	Negative	Negative	Negative
BT 248	Negative	Negative	Negative	Negative
BT 249	Negative	Negative	Negative	Negative
BT 250	Negative	Negative	Negative	Negative

BT 251	Negative	Negative	Negative	Negative
BT 252	Negative	Negative	Negative	Negative
BT 253	Negative	Negative	Negative	Negative
BT 254	Negative	Negative	Negative	Negative
BT 255	Positive	Negative	Negative	Negative
BT 256	Negative	Negative	Negative	Negative
BT 257	Negative	Negative	Negative	Negative
BT 258	Negative	Negative	Negative	Negative
BT 259	Negative	Negative	Negative	Negative
BT 260	Positive	Negative	Positive	Positive
BT 261	Negative	Negative	Negative	Negative
BT 262	Negative	Negative	Negative	Negative
BT 263	Negative	Negative	Negative	Negative
BT 264	Negative	Negative	Negative	Negative
BT 265	Negative	Negative	Negative	Negative
BT 266	Negative	Negative	Negative	Negative
BT 267	Negative	Negative	Negative	Negative
BT 268	Negative	Negative	Negative	Negative
BT 269	Positive	Negative	Positive	Positive
BT 270	Negative	Negative	Negative	Negative
BT 271	Negative	Negative	Negative	Negative
BT 272	Negative	Negative	Negative	Negative
BT 273	Negative	Negative	Negative	Negative
BT 274	Negative	Negative	Positive	Negative
BT 275	Positive	Positive	Positive	Positive
BT 276	Positive	Negative	Positive	Positive
BT 277	Negative	Negative	Positive	Negative
BT 278	Negative	Negative	Positive	Negative
BT 279	Negative	Negative	Positive	Positive
BT 280	Negative	Negative	Positive	Positive
BT 281	Negative	Negative	Positive	Positive
BT 282	Negative	Negative	Positive	Positive
BT 283	Negative	Negative	Positive	Positive
BT 284	Negative	Negative	Positive	Positive

BT 285	Positive	Negative	Positive	Negative
BT 286	Positive	Negative	Positive	Negative
BT 287	Positive	Positive	Positive	Positive
BT 288	Positive	Negative	Positive	Positive

Sample ID	Phenotypic DST	Phenotypic DST	Xpert MTB/RIF	Abbott RealTime RIF/INH	
	**Rifampicin**	**Isoniazid**	**Rifampicin**	**Rifampicin**	**Isoniazid**
BT 001	Resistant	Resistant	Resistant	Resistant	Resistant
BT 003	Susceptible	Susceptible	Susceptible	Susceptible	Susceptible
BT 004	Resistant	Resistant	Resistant	Resistant	Resistant
BT 007	Susceptible	Susceptible	Susceptible	Susceptible	Susceptible
BT 008	Resistant	Resistant	Resistant	Resistant	Resistant
BT 017	Susceptible	Susceptible	Susceptible	Susceptible	Susceptible
BT 018	Susceptible	Susceptible	Susceptible	Susceptible	Susceptible
BT 021	Susceptible	Susceptible	Susceptible	Susceptible	Susceptible
BT 028	Resistant	Resistant	Resistant	Resistant	Resistant
BT 036	Resistant	Resistant	Resistant	Resistant	Resistant
BT 039	Susceptible	Susceptible	Susceptible	Susceptible	Susceptible
BT 040	Resistant	Resistant	Resistant	Resistant	Resistant
BT 045	Susceptible	Susceptible	Susceptible	Susceptible	Susceptible
BT 046	Susceptible	Susceptible	Susceptible	Susceptible	Susceptible
BT 049	Susceptible	Susceptible	Susceptible	Susceptible	Susceptible
BT 052	Resistant	Resistant	Resistant	Resistant	Susceptible
BT 058	Susceptible	Susceptible	Susceptible	Susceptible	Susceptible
BT 059	Susceptible	Susceptible	Susceptible	Susceptible	Susceptible
BT 060	Susceptible	Susceptible	Susceptible	Susceptible	Susceptible
BT 064	Resistant	Resistant	Resistant	Resistant	Resistant
BT 065	Susceptible	Susceptible	Susceptible	Susceptible	Susceptible
BT 069	Susceptible	Susceptible	Susceptible	Susceptible	Susceptible
BT 073	Susceptible	Susceptible	Susceptible	Susceptible	Susceptible
BT 074	Susceptible	Susceptible	Susceptible	Susceptible	Susceptible
BT 077	Susceptible	Susceptible	Susceptible	Susceptible	Susceptible
BT 082	Resistant	Resistant	Resistant	Resistant	Susceptible
BT 084	Susceptible	Susceptible	Susceptible	Susceptible	Susceptible
BT 086	Susceptible	Susceptible	Susceptible	Susceptible	Susceptible
BT 087	Susceptible	Susceptible	Susceptible	Susceptible	Susceptible
BT 092	Resistant	Resistant	Resistant	Resistant	Resistant
BT 096	Susceptible	Susceptible	Susceptible	Susceptible	Susceptible
BT 103	Susceptible	Resistant	Susceptible	Susceptible	Resistant
BT 112	Susceptible	Resistant	Susceptible	Susceptible	Resistant
BT 119	Susceptible	Resistant	Susceptible	Susceptible	Resistant
BT 120	Resistant	Susceptible	Resistant	Resistant	Susceptible
BT 125	Susceptible	Susceptible	Susceptible	Susceptible	Susceptible
BT 147	Susceptible	Susceptible	Susceptible	Susceptible	Susceptible
BT 148	Susceptible	Resistant	Susceptible	Susceptible	Resistant
BT 151	Susceptible	Resistant	Susceptible	Susceptible	Resistant
BT 152	Susceptible	Susceptible	Susceptible	Susceptible	Susceptible
BT 167	Susceptible	Susceptible	Susceptible	Susceptible	Susceptible
BT 183	Susceptible	Susceptible	Susceptible	Susceptible	Susceptible
BT 189	Susceptible	Resistant	Susceptible	Susceptible	Resistant
BT 190	Susceptible	Susceptible	Susceptible	Susceptible	Susceptible

BT 191	Susceptible	Susceptible	Susceptible	Susceptible	Susceptible
BT 195	Susceptible	Resistant	Susceptible	Susceptible	Resistant
BT 201	Susceptible	Resistant	Susceptible	Susceptible	Susceptible
BT 246	Susceptible	Susceptible	Susceptible	Susceptible	Susceptible
BT 255	Susceptible	Susceptible	Susceptible	Susceptible	Susceptible
BT 261	Susceptible	Susceptible	Susceptible	Susceptible	Susceptible
BT 262	Susceptible	Susceptible	Susceptible	Susceptible	Susceptible
BT 273	Susceptible	Resistant	Susceptible	Susceptible	Resistant
BT 274	Resistant	Susceptible	Resistant	Resistant	Susceptible
